# Physicochemical property and microbial community characteristics of the casing soil for cultivating *Oudemansiella raphanipes*

**DOI:** 10.3389/fmicb.2024.1495168

**Published:** 2024-12-05

**Authors:** Jinjia Liu, Zhongyu Qin, Jinqiang Wu, Jiao Su, Pengcheng Feng, Wenting Su

**Affiliations:** ^1^Department of Biochemistry, Changzhi Medical College, Changzhi City, Shanxi Province, China; ^2^College of Animal Science, Shanxi Agriculture University, Taigu, Jinzhong City, Shanxi Province, China; ^3^Department of Histology and Embryology, Changzhi Medical College, Changzhi City, Shanxi Province, China; ^4^Department of Physiology, Changzhi Medical College, Changzhi City, Shanxi Province, China

**Keywords:** *Oudemansiella raphanipes*, soil, enzyme activity, physicochemical property, soil microorganisms

## Abstract

**Background:**

Casing soil is critical for the cultivation process of *Oudemansiella raphanipes* and promotes the formation of mushroom fruiting bodies. Therefore, reliable casing soil indicators are crucial for obtaining high yields of high-quality mushrooms.

**Methods:**

In this study, soil enzyme activity, physicochemical properties, and microorganisms at five cultivation stages [namely casing (A1), mycelial (A2), primordial (A3), fruiting (A4), and harvesting (A5)] of *O. raphanipes* cultivation were evaluated in casing soils.

**Results:**

The results indicated that sucrase and catalase activities were significantly increased with increasing cultivation time (*p* < 0.01), and the activities peaked [16.67 and 0.25 g/(g·h), respectively] at A4. Urease activity peaked [1.56 g/(g·h)] at A1, and it decreased gradually (*p* < 0.01). Polyphenol oxidase activity was significantly higher at A2 [0.95 g/(g·h)] than at the other stages and was significantly lower at A1 [0.06 g/(g·h)]. Soil pH peaked at A1 (8.20) and decreased gradually (*p* = 0.003). Soil total organic carbon content increased significantly with increasing cultivation time (*p* < 0.001) and was the highest at A5 (8.40 g/kg). The available phosphorus at A1 (0.40 g/kg) was significantly higher than those at the other stages (*p* = 0.004), and the available nitrogen at A1 (0.28 g/kg) and A3 (0.26 g/kg) was significantly higher than those at the other stages (*p* < 0.001). The number and diversity of bacteria and fungi in soil increased gradually, and nine bacterial and four fungal genera were identified.

**Conclusion:**

This study offers soil characteristic and microbial community data for *O. raphanipes* casing soil at different cultivation stages, which could facilitate sustainable cultivation of *O. raphanipes* and reduction of live contaminants.

## 1 Introduction

*Oudemansiella raphanipes* is a fungus belonging to phylum Basidiomycota, and commonly known as “Heipijizong” or “Changgengu” in China. It exhibits high palatability, unique mouthfeel, and richness in various bioactive components, such as proteins, amino acids, bioenzymes, polyphenols, flavonoids, and polysaccharides (Hao et al., 2016). In addition, *O. raphanipes* provides hypotensive and tumor-inhibiting effects, making it a popular fungus with a rare dietary-medicinal dual purpose (Gao et al., 2018; Wu et al., 2019). Statistics from the China Edible Fungi Association indicate that the total production of *O. raphanipes* in China reached 19,502 tons in 2020.

Cultivation under a casing soil (casing cultivation) is a common approach used in *O. raphanipes* production. By covering the fungal bed with a layer of pre-treated casing soil during the cultivation process, the microenvironmental conditions, such as temperature, light, water, and air on the surface of the cultivation substrate, are altered. This provides the essential environment for the growth stage transition (vegetative to reproductive). Soil physicochemical properties play a crucial role in influencing soil microorganisms and undergo certain changes with the growth of fruiting bodies. This affects the diversity and structure of microbial communities, ultimately influencing fruiting body yield and quality (McGee, 2018; Pecchia et al., 2014). Casing cultivation has become the primary mode of *O. raphanipes* production, as it provides good fruiting effects with intact fruiting body shapes. Loam soil is selected as the casing soil owing to its high air permeability and water-holding capacity (Xiao et al., 2022). Other requirements of casing soils include appropriate particle size and porosity, good aggregate structure, and high microbial diversity (Huang et al., 2022).

The most prominent challenge associated with fruiting in casing soils is contamination by miscellaneous microorganisms originating from the soil. Inadequate soil sterilization leads to the introduction of pathogenic microorganisms during casing, which usually causes *O. raphanipes* infection and results in huge production losses. Previous studies have indicated that the fruiting body yields of *Agaricus bisporus* and *Ganoderma lucidum* are significantly correlated with fungal community diversity in the casing soil (Cai et al., 2009; Ke et al., 2019; Ren et al., 2020). In the *A. bisporus* cultivation system, native microorganisms and fungi coexist and interact in a symbiotic relationship. Studies have shown that early fruiting body formation may be related to changes in bacterial communities. During cultivation, some bacterial structures undergo continuous changes and tend to stabilize, with *Pseudomonas* spp. being potentially significant in the formation of *A. bisporus* fruiting bodies (Vieira et al., 2023). A study on the casing soil for cultivating *Ganoderma lingzhi* revealed that the fungal diversity in the casing soil decreased gradually (Yuan et al., 2019). In contrast, an increase was observed in the relative abundance of the fungi belonging to the genus *Penicillium*, a crucial fungal group that hinders continuous cropping in the local region. The market demand for *O. raphanipes* has shown a steady increase recently. Therefore, investigating the physicochemical properties and microbial community structures of casing soils and identifying optimal ecological environments for *O. raphanipes* growth, particularly the soil environment during casing cultivation, are of essential theoretical and practical significance for elucidating the microecological mechanisms in the casing soil of *O. raphanipes*, improving mushroom yield and overcoming the limitations in cultivation techniques.

The aim of this study was to investigate soil enzyme activity, physicochemical properties, and microbial community structure at different casing cultivation stages of *O. raphanipes*. It was speculated that the physicochemical properties of soil enzyme activity would vary in different cultivation stages and that the number and types of microorganisms would increase gradually during cultivation. Soil physicochemical properties and microbial community structure are possibly related. Some microorganisms might have positive or negative effects on *O. raphanipes* cultivation. Our results can establish a theoretical foundation for the scientific and rational cultivation of *O. raphanipes*, reducing contamination from mixed bacteria during the cultivation process to help growers design programs to control the development of *O. raphanipes*., and laying the groundwork for *promoting* large-scale production of *O. raphanipes*.

## 2 Materials and methods

### 2.1 Experimental design and soil sample collection

An *O. raphanipes* casing cultivation experiment was conducted in the greenhouse of an edible fungus farm in Xiaodian District, Taiyuan City, Shanxi Province of China, in May 2020. The fungal strains were provided by the Shanxi Institute of Biology Co., Ltd. Loam soil with a casing thickness of 4–5 cm was used as the casing soil. After casing, watering was performed once every 5 days, and the temperature and air humidity within the greenhouse were maintained at 25°C and > 90%, respectively. Shading and cooling were strictly monitored during the cultivation management process. Samples were collected using the five-point sampling method during five cultivation stages, including Days 0, 15, 25, 40, and 50, representing the casing (A1), mycelial (A2), primordial (A3), fruiting (A4), and harvesting (A5) stages, respectively. The loam soil sample was collected at a 2–4 cm depth after removing the surface soil. The five-point sampling method was consistently adopted for all casing soil samples. Soil samples from five sampling points were randomly selected, and 100 g of soil from each sampling point was obtained and mixed to form a composite sample. Three replicates were established for each growth stage, resulting in 15 samples. Following the removal of large particles using a 2-mm sieve, samples were quickly placed in sterile glass containers, returned to the laboratory in an ice box, and stored at −80°C.

### 2.2 Measurement of soil enzymatic activities

Sucrase, urease, catalase, and polyphenol oxidase activities in the soil were evaluated using the 3,5-dinitrosalicylic acid colorimetric, phenol-sodium hypochlorite colorimetric, potassium permanganate titration, and pyrogallol colorimetric methods, respectively. The measurements were performed following the procedures described by Song et al. (2023).

### 2.3 Measurement of soil physicochemical properties

The pH of the soil samples was measured using a pH meter (soil/water mass ratio of 1:2.5). The total organic carbon (TOC) content was determined using the potassium dichromate oxidation-external heating method (Ding and Fang, 2024). The available nitrogen (AN) content was measured using the alkaline hydrolysis diffusion method (Yu et al., 2024), and the available phosphorus (AP) content was determined through ultraviolet spectrophotometry (Liu et al., 2023).

### 2.4 Isolation and counting of soil microorganisms

Counts of the major groups of soil microorganisms were determined using the dilution spread plate method (Xu et al., 1984). Bacteria and fungi were separately cultured on beef extract-peptone (Wang et al., 2017) and Martin’s media (Juan et al., 2020). Species accumulation curves for bacteria and fungi were derived. The expected species richness (S_p_) was estimated per the jackknife1 procedure (Heltshe and Forrester, 1983) using the following formula:


$$S_{p}=S_{o}+\frac{f_{1}\left(N-1\right)}{N}$$

*S_o_* is the observed number of species, *f_1_* is the number of species occurring in only one sample plot, and *N* is the number of sample plots.

To obtain *S_p_*, species accumulation rate analysis was conducted for each plot, and the species accumulation curves were generated from the result of 100 random accumulations using the Vegan package in R version 2.14.0 (Li, 2011).

### 2.5 DNA extraction, amplification, and sequencing of soil microorganisms

Genomic DNA was extracted from soil samples using the E.Z.N.A.® Soil DNA kit (Omega Bio-Tek) following the manufacturer’s instructions. The V4 region of the 18S ribosomal RNA (rRNA) gene was amplified through PCR using the barcoded primers 528F (5′-GCGGTAATTCCAGCTCCAA-3′) and 706R (5′-AATCCRAGA ATTTCACCTCT-3′) and the amplification reaction system and procedure described by Zhang et al. (2023). The V3–V4 regions of bacterial 16S rRNA genes in the samples were amplified through PCR using the primers 338F (5′-ACTCCTACGGGAGGCAGCA-3′) and 806R (5′-GACTACHVGGGTWTCTAAT-3′) and the amplification reaction system and procedure described by Wang et al. (2024). The PCR products were purified using the AxyPrep DNA Gel Extraction Kit (Axygen Biosciences) and subsequently subjected to NovaSeq sequencing by Guangzhou IGE Bio-technology Co., Ltd.

### 2.6 Data analysis

Soil enzyme activity, physicochemical properties, and microbial population data were analyzed through a one-way analysis of variance using IBM SPSS Statistics 20 (IBM Corp.), and the significant differences were tested using the multiple comparison method (Duncan). For the composition of operational taxonomic units among microbial species identified from the sequencing, raw sequences for bacterial 16S rRNA and fungal 18S rRNA genes that had passed quality pre-screening were sorted into libraries and samples based on index and barcode information. Barcode sequences and primers were removed. High-quality tag data obtained using the tag quality control process of the QIIME2 system (Bolyen et al., 2019) were the effective tag data. Species annotation was performed using Ribosomal Database Project Classifier version 2.2, and the Silva database (Release 138, http://www.arbsilva.de) was used to determine the bacterial and fungal compositions at the phylum and genus levels in each sample (Zhang et al., 2023).

Principal coordinate analysis (PCoA) based on the Bray–Curtis distance was used to determine structural differences between bacterial and fungal communities in the casing soil. Phyloseq and ggplot2 R packages (3.3.6) were used to perform principal coordinate analysis and make PCoA plots. Redundancy Analysis (RDA) was conducted to analyze the effects of soil physicochemical properties on microbial communities. Enfit test was used to estimate the significant correlation of multiple relationships in the RDA plot, and the threshold was set as *r*^2^ > 0.5, *p* < 0.01 (Tan et al., 2022). Based on the species annotation and abundance information of all samples at each classification level, clustering was conducted according to their abundance information in each sample. The igraph program package in R combined with Gephi software was used to analyze cooccurrence network (Tan et al., 2022).

## 3 Results

### 3.1 Soil enzymatic activity at different cultivation stages of *Oudemansiella raphanipes*

**Figure 1:**
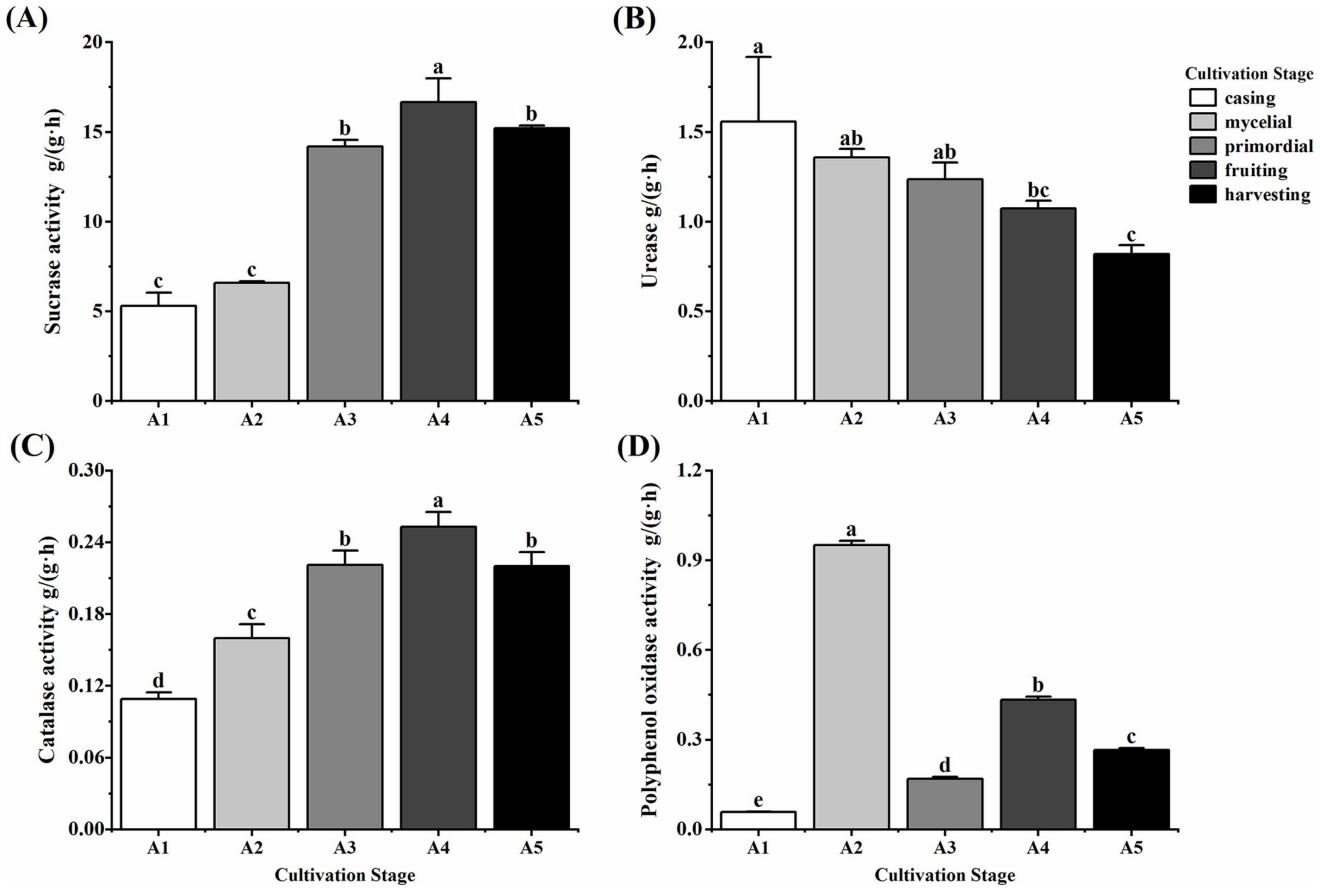
Soil enzyme activity in different cultivation stages of *Oudemansiella raphanipes.* **(A)** Sucrase activity of casing soil across five cultivation stages; **(B)** Urease activity of casing soil across five cultivation stages; **(C)** Catalase activity of casing soil across five cultivation stages; **(D)** Polyphenol oxidase activity of casing soil across five cultivation stages.

Soil enzyme activity varied significantly among the five casing cultivation stages of *O. raphanipes* (Figure 1). Sucrase activity increased significantly during the cultivation process (*F* = 166.88, df = 4, *p* < 0.001), peaking at 16.67 g/(g·h) at A4 and decreasing slightly subsequently (Figure 1A). Soil urease activity decreased significantly from 1.56 g/(g·h) at A1 to 0.82 g/(g·h) at A5 (*F* = 8.07, df = 4, *p* = 0.004) (Figure 1B). Catalase activity increased gradually from 0.11 g/(g·h) at A1 to 0.25 g/(g·h) at A4 and decreased significantly to 0.22 g/(g·h) at A5 (*F* = 82.72, df = 4, *p* < 0.001) (Figure 1C). Polyphenol oxidase activity showed no clear trend (*F* = 4653.46, df = 4, *p* < 0.001), exhibiting a low level of 0.06 g/(g·h) at A1, a rapid increase to the first peak of 0.95 g/(g·h) at A2, a decrease with extensive mycelial growth, an increase to a second peak of 0.43 g/(g·h) at A4, and finally a decrease to 0.27 g/(g·h) at A5 (Figure 1D).

### 3.2 Soil physicochemical indicators at different cultivation stages of *Oudemansiella raphanipes*

**Figure 2:**
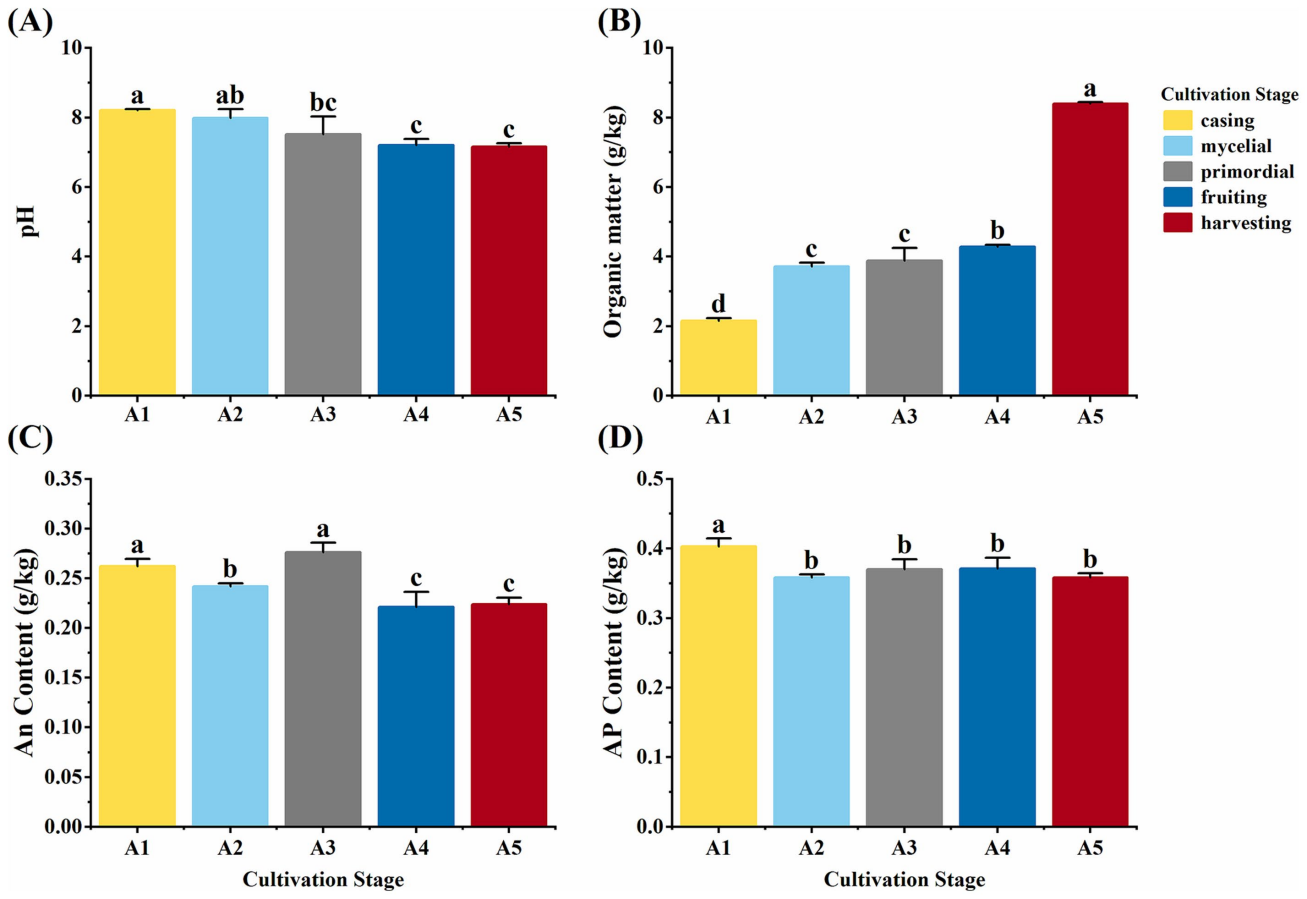
Soil physicochemical properties in different cultivation stages of *Oudemansiella raphanipes.* **(A)** Casing soil pH across five cultivation stages; **(B)** Soil total organic carbon content across five cultivation stages; **(C)** Available nitrogen (AN) content across five cultivation stages; **(D)** AP content across five cultivation stages.

Various soil physicochemical properties were assessed at different casing cultivation stages of *O. raphanipes*. Soil pH decreased significantly from 8.20 at A1 to 7.52 at A3 and showed no significant changes subsequently (*F* = 8.86, df = 4, *p* = 0.003) (Figure 2A). Soil TOC content was increased significantly from 2.15 g/kg at A1 to 8.40 g/kg at A5 (*F* = 506.42, df = 4, *p* < 0.001) (Figure 2B). AN content was 0.28 g/kg at the A3 stage and 0.26 g/kg at the A1 stage, which were significantly higher than those at the other stages (A2: 0.24 g/kg; A4: 0.22 g/kg; A5: 0.22 g/kg) (*F* = 19.27, df = 4, *p* < 0.001) (Figure 2C). Conversely, AP content was decreased significantly from 0.40 g/kg at A1 to 0.36 g/kg at A2 and subsequently remained stable with no significant changes (*F* = 7.80, df = 4, *p* = 0.004) (Figure 2D).

### 3.3 Soil microorganism levels at different cultivation stages of *Oudemansiella raphanipes*

**Figure 3:**
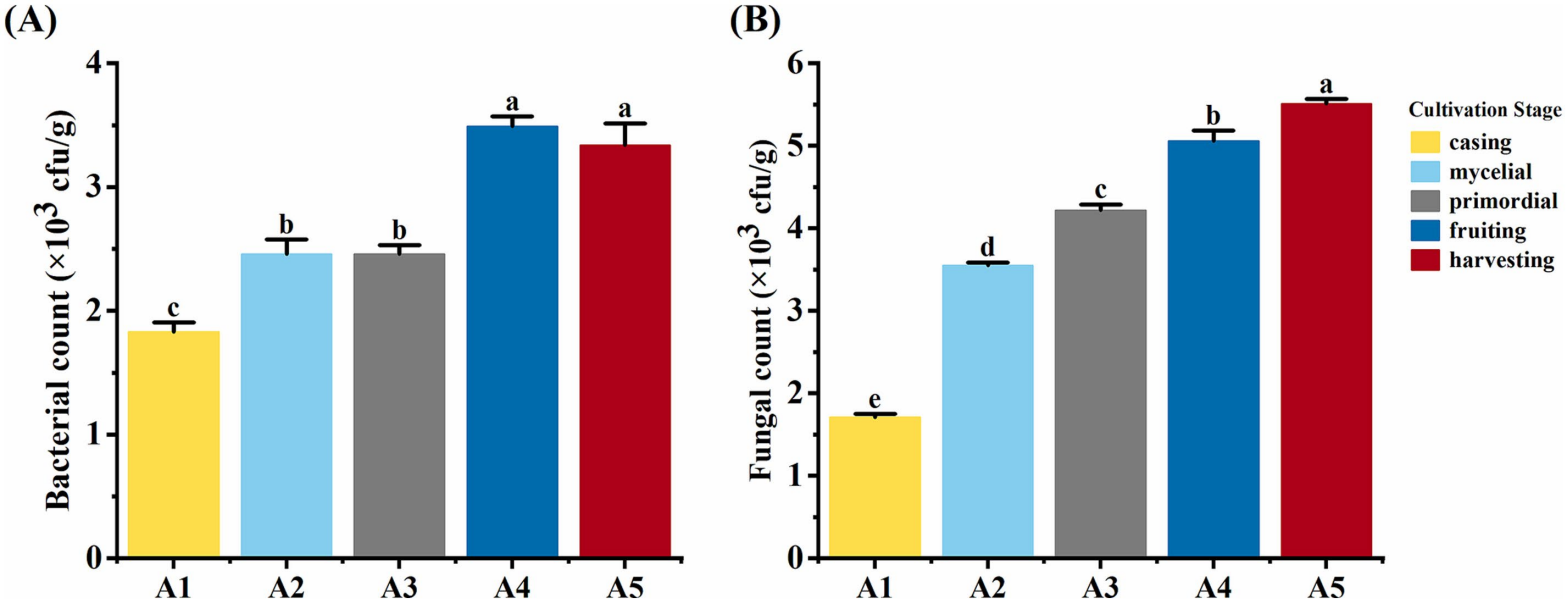
Soil microorganism numbers at different cultivation stages of *Oudemansiella raphanipes.* **(A)** Bacteria number in casing soil across five cultivation stages; **(B)** Fungi number in casing soil across five cultivation stages.

With the progression of the casing cultivation of *O. raphanipes*, soil bacterial count showed an initial increase, stabilization, further increase, and final stabilization (*F* = 117.85, df = 4, *p* < 0.001). Bacterial count increased significantly from 1.8 × 10^3^ cfu/g at A1 to 2.4 × 10^3^ cfu/g at A2, further increasing significantly from 2.4 × 10^3^ cfu/g at A3 to 3.5 × 10^3^ cfu/g at A4, and stabilized at 3.3 × 10^3^ cfu/g (Figure 3A). Soil fungal count increased significantly from 1.71 × 10^3^ cfu/g at A1 to 5.06 × 10^3^ cfu/g at A4 and showed no significant changes from A4 to A5 (*F* = 1283.40, df = 4, *p* < 0.001) (Figure 3B).

**Figure 4:**
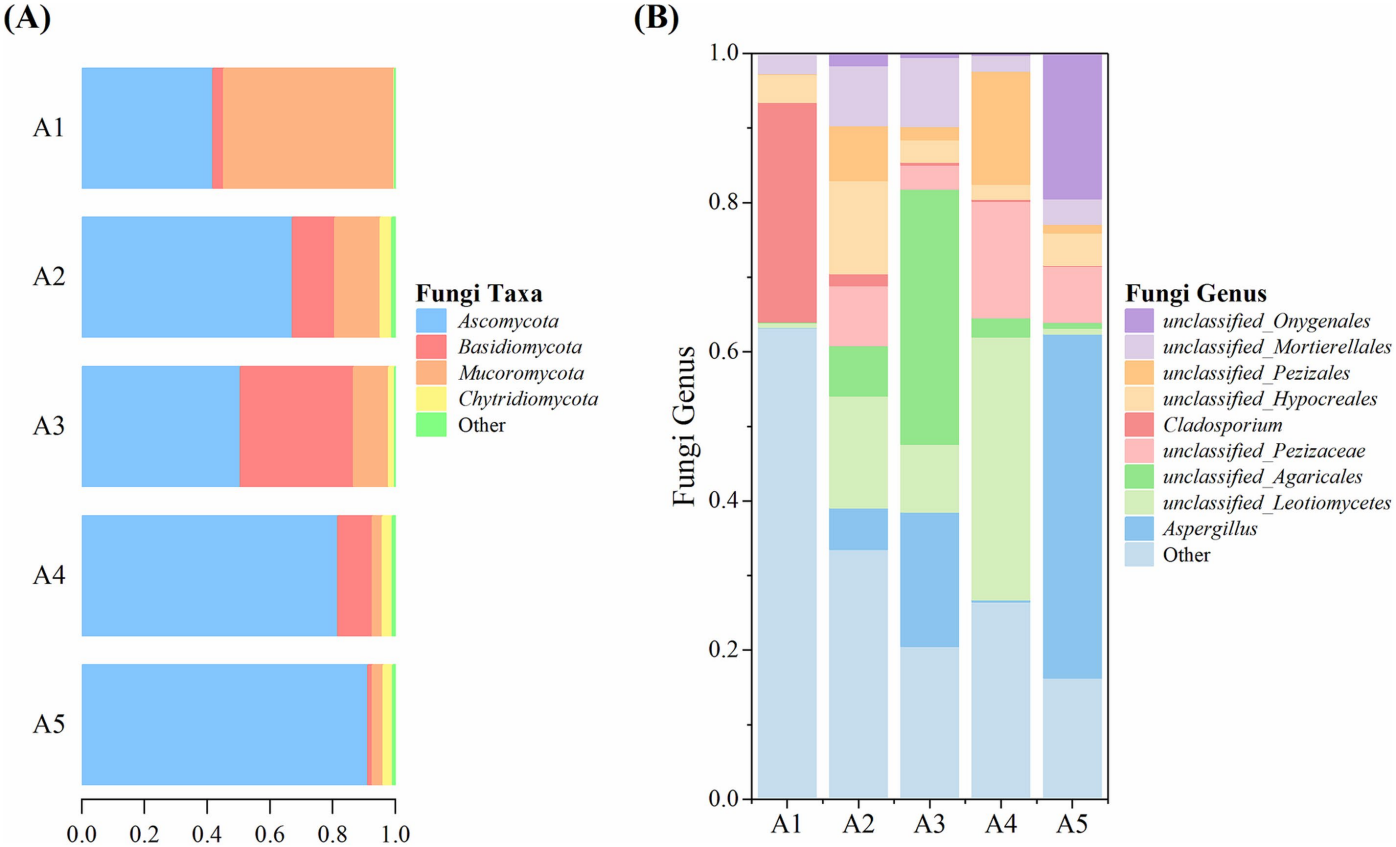
Sample-based species accumulation curves. **(A)** Bacterial accumulation curve; **(B)** Fungal accumulation curve.

Species accumulation curves are used to describe increasing trends of species with an increase in sampling effort and aid in investigating species composition in sample plots and predicting species richness. The species accumulation curves for soil bacteria and fungi (Figure 4) showed a steep increase when the sample size was small. With an increase in the sample size, the significant increase in the total number of bacterial and fungal species stopped owing to the addition of new samples. The curves leveled off gradually and became asymptotic, indicating that sampling was sufficient for data analysis.

### 3.4 Soil microbial community structure and composition

#### 3.4.1 *β*-Diversity analysis of soil microorganisms

**Figure 5:**
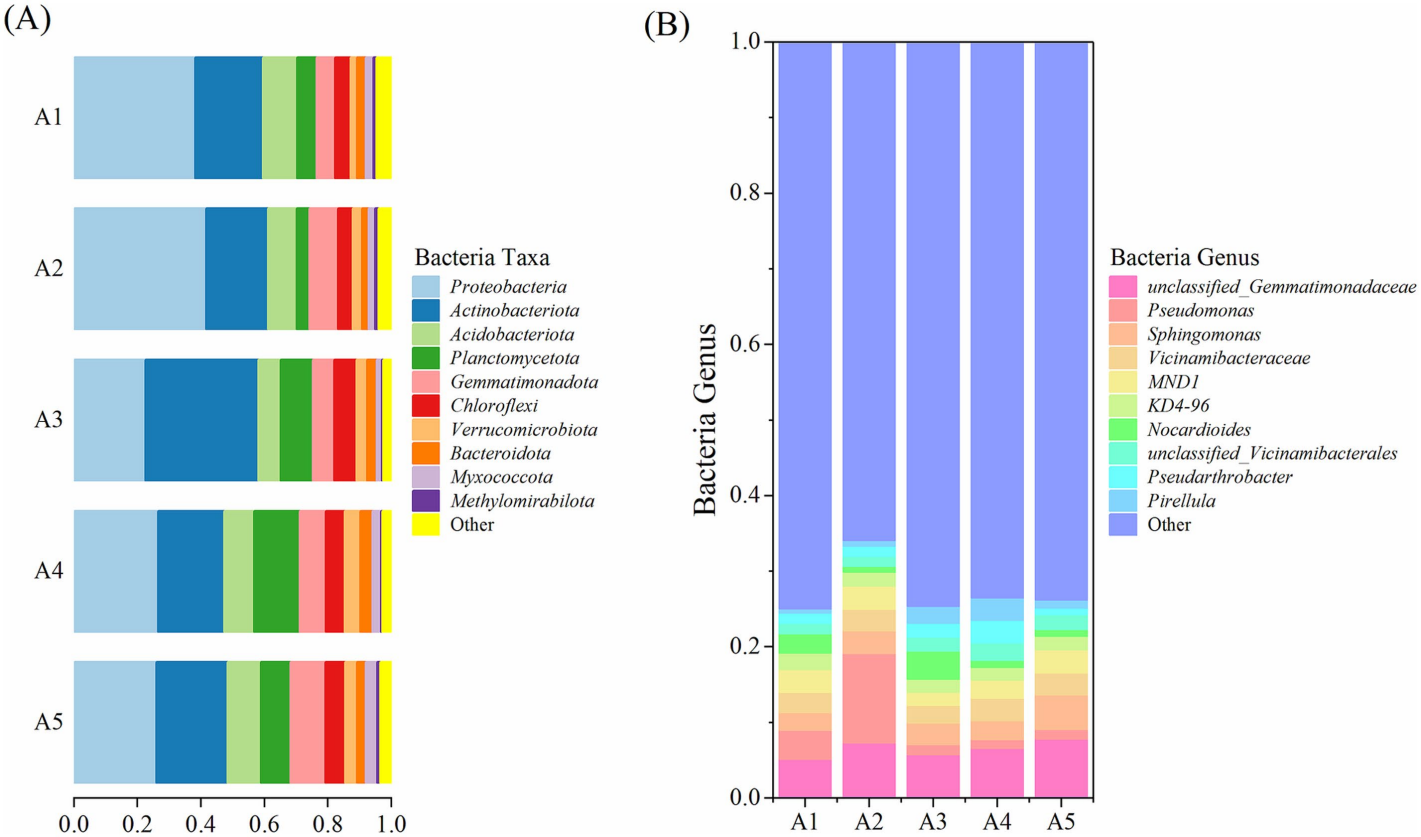
Structure analysis of the microbial community in casing soil cultivation of *Oudemansiella raphanipes* using Principal Component Analysis (PCoA). **(A)** Bacterial community structure; **(B)** Fungal community structure.

A β-diversity analysis of the casing soil bacterial community structure before and after *O. raphanipes* cultivation was conducted using PCoA based on the Bray–Curtis distance (Figure 5). Results showed that soil samples A3 and A4 of *O. raphanipes* were clustered together along the horizontal (57.55% explained variance) and vertical (18.16% explained variance) axes and were dispersed from other samples, indicating that the bacterial community structures in the soil during the primordium and fruiting stages of *O. raphanipes* were similar. However, the bacterial communities of other samples were dispersed across the horizontal and vertical axes of PCoA, indicating significant differences in the bacterial community structure of other casing soil samples (Figure 5A).

The β-diversity analysis of soil fungal community structure showed that soil samples A2, A3, and A4 were clustered together along the horizontal (42.53% explained variance) and vertical (31.9% explained variance) axes and were dispersed from other samples, indicating similar fungal community structures in the soil during the mycelial, primordial, and fruiting stages of *O. raphanipes* cultivation. However, the fungal communities in other samples dispersed across the horizontal and vertical axes of PCoA, demonstrating significant differences in the fungal community structures among these samples (Figure 5B).

#### 3.4.2 Redundancy analysis of soil microorganisms

Redundancy analysis was conducted using casing soil pH, TOC, AN, and AP to determine the impact of soil physicochemical properties on bacterial and fungal communities during the cultivation of *O. raphanipes*. Each point in the resulting graph represents a different sample, arrows starting from the origin represent different physical and chemical properties: the length of the arrow represented the intensity of the effect of the physicochemical property on the community change; the Angle between the arrow and the coordinate axis represented the correlation between the physical and chemical properties and the coordinate axis; the distance from the sample point to the arrow indicated the strength of the physical and chemical properties on the sample.

**Figure 6:**
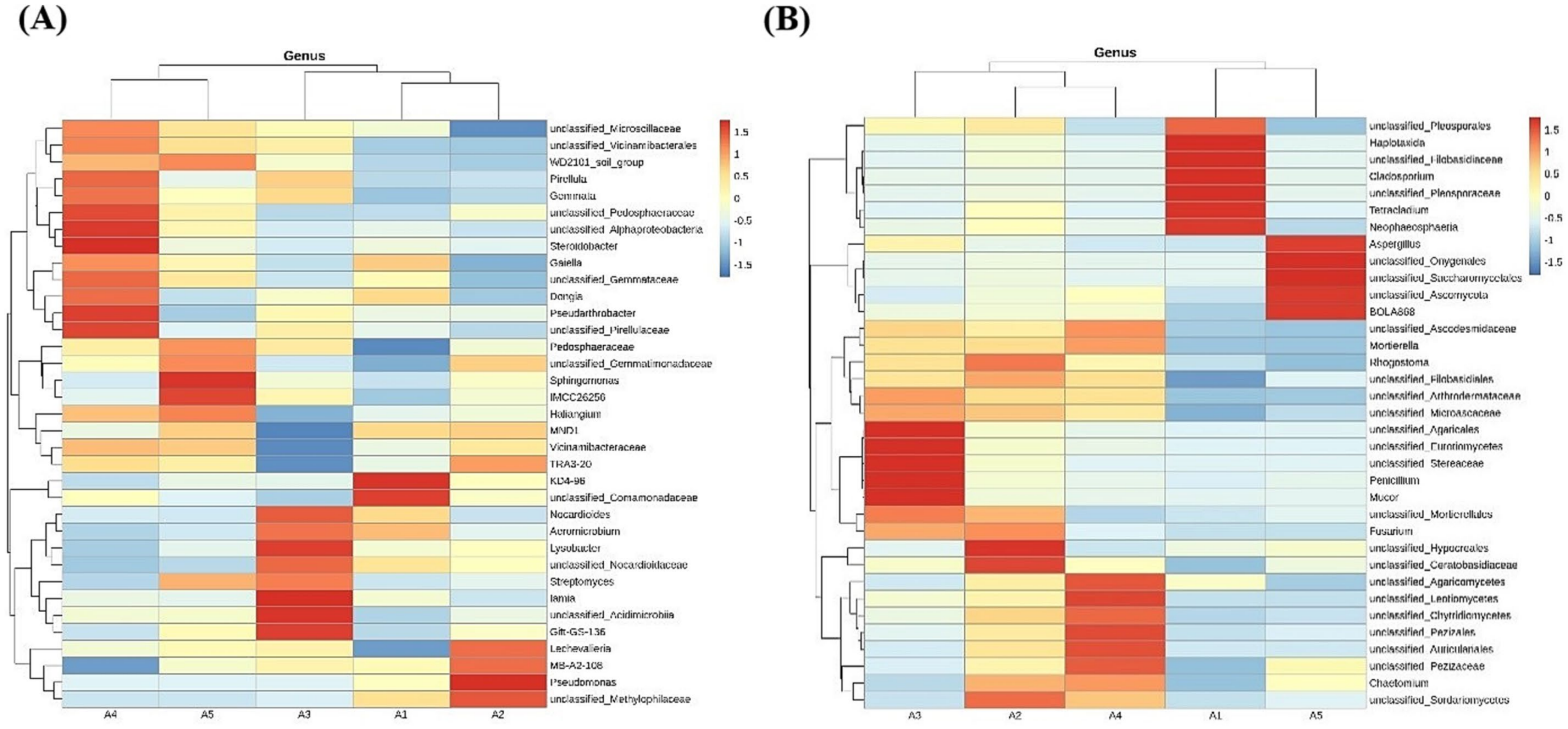
Effect of the physicochemical properties of the casing soil on microbial communities. **(A)** Effects of the physicochemical properties on bacterial communities. **(B)** Effects of the physicochemical properties on fungal communities. OM, Organic matter; TN, Total nitrogen; AP, Available phosphorus; AK, Available potassium.

The results indicated that organic matter showed the largest influence on the change of bacterial community. The TOC had significant influence on the bacterial community of samples A4 and A5 and was positively correlated. The pH, AP and AN had significant effect on the bacterial community of sample A1 and were positively correlated. The total explanatory power of these factors for bacterial community difference before and after *O. raphanipes* cultivation was 56.02% (Figure 6A).

Similarly, RDA of fungal community showed that soil pH, AP, and AN affected the fungal community of sample A1 and showed a positive correlation, in which pH had the most significant effect on the fungal community of sample A1. The TOC was positively correlated with the changes of soil fungal communities in A3, A4, and A5, and significantly affected the fungal communities in samples A5 and A4. The total explanatory power of these factors for the difference of fungal community before and after *O. raphanipes* cultivation was 36.10% (Figure 6B).

#### 3.4.3 Microbial community composition of soil microorganisms

**Figure 7:**
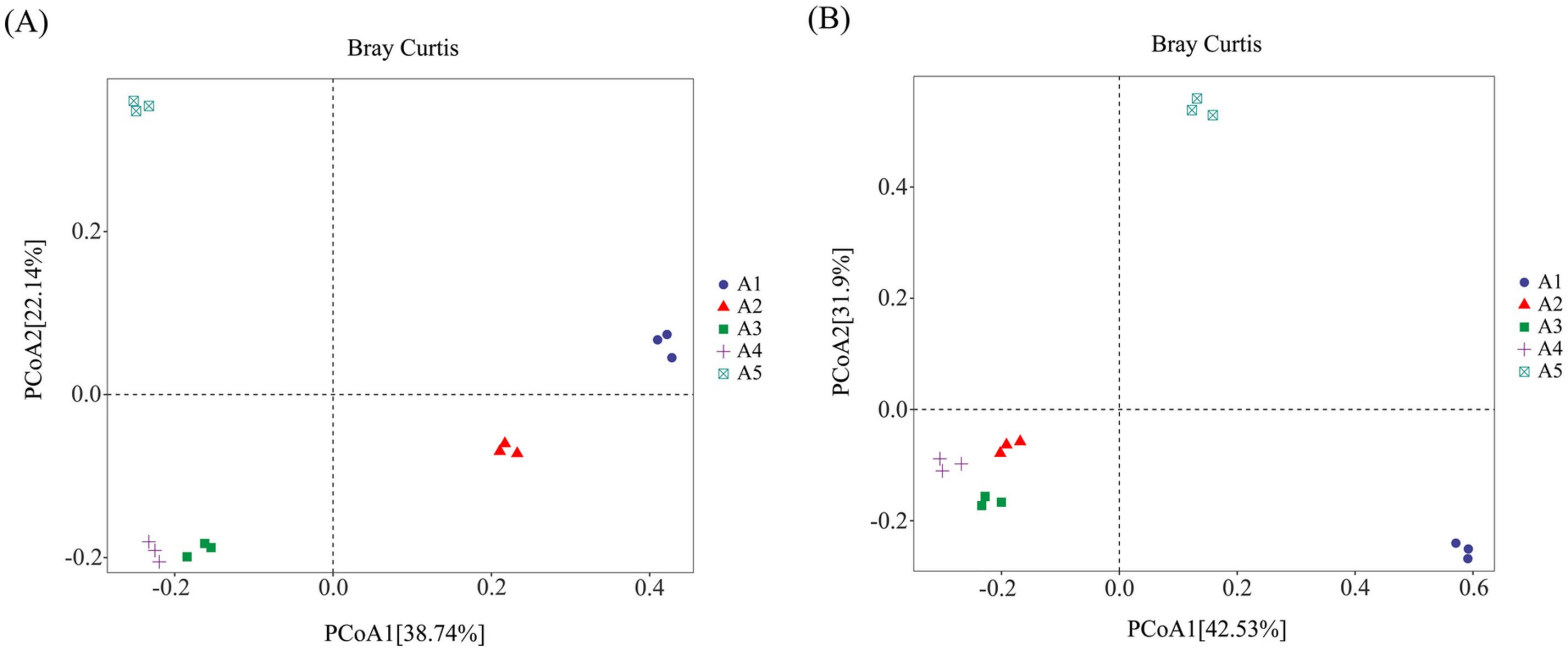
Bacterial taxa and genera in the soil at different cultivation stages of *Oudemansiella raphanipes.* **(A)** Identified bacterial taxa in casing soil; **(B)** Identified bacterial genera in casing soil.

The top 10 bacterial phyla in the five soil samples determined using high-throughput sequencing were *Proteobacteria*, *Actinobacteriota*, *Acidobacteriota*, *Planctomycetota*, *Gemmatimonadota*, *Chloroflexi*, *Verrucomicrobiota*, *Bacteroidota*, *Myxococcota*, and *Methylomirabilota* (Figure 7A). *Proteobacteria* and *Actinobacteriota* showed absolute dominance, with relative abundances of 22.47–41.62% and 19.45–35.59%, respectively, at different casing cultivation stages of *O. raphanipes*. These were followed by *Acidobacteriota*, *Planctomycetota*, *Gemmatimonadota*, and *Chloroflexi*, with relative abundances of 3.91–14.35%, and subsequently by *Verrucomicrobiota*, *Bacteroidota*, *Myxococcota*, and *Methylomirabilota*, with relative abundances of 0.34–4.94%.

At the genus level, an unclassified genus in the family *Gemmatimonadaceae* exhibited the highest abundance, with a relative abundance of 7.83% at A4 (Figure 7B). *Pseudomonas* had a relative abundance of 1.16–11.85%, with abundance at A2 being significantly higher than those at other stages. The relative abundances of other classified bacterial genera were < 5% (*Sphingomonas*: 2.38–4.56%, *Vicinamibacteraceae*: 2.33–2.95%, *Nocardioides*: 0.81–3.72%, an unclassified bacterial genus of the family *Vicinamibacteraceae*: 1.28–2.26%, fungi of the genus *MND*1: 1.73–3.07%, fungi of the genus *KD*4-96: 1.66–2.17%, *Pseudarthrobacter*: 0.91–3.02%, and *Pirellula*: 0.6–2.97%). The relative abundances of other bacterial genera ranged from 66.0 to 74.93%.

**Figure 8:**
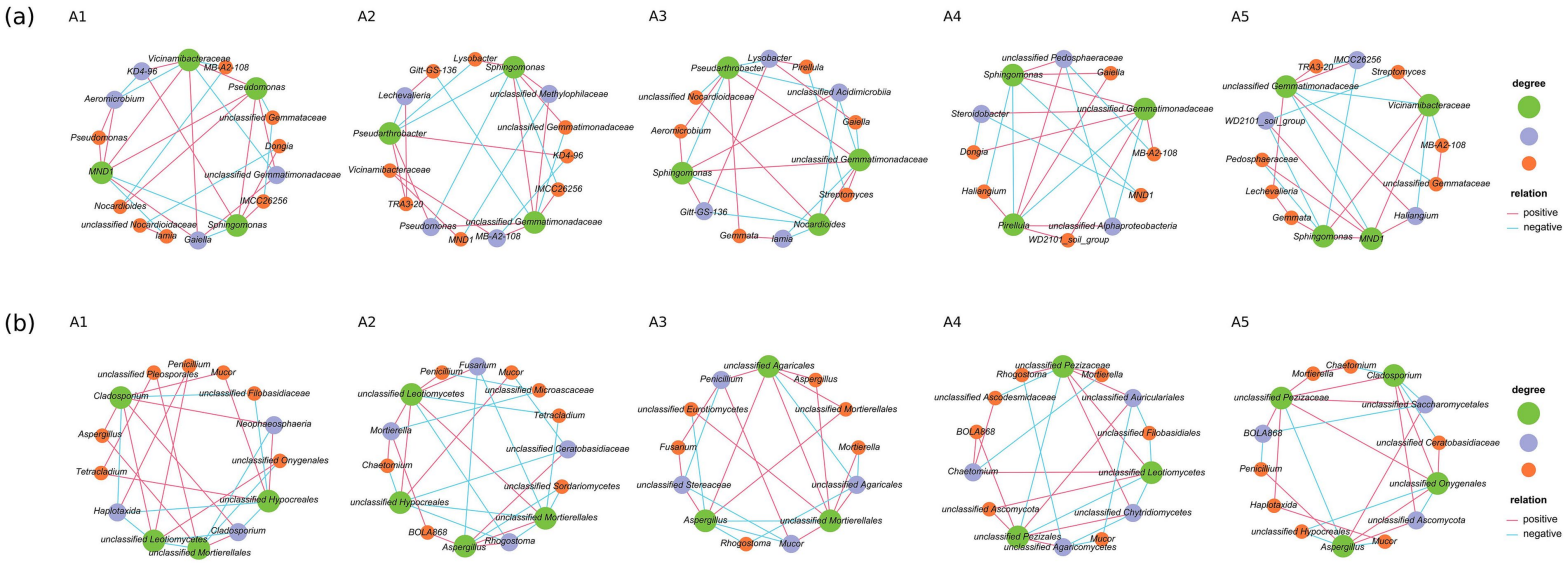
Fungal taxa and genera in the soil at different cultivation stages of *Oudemansiella raphanipes.* **(A)** Identified fungal taxa in casing soil; **(B)** Identified fungal genera in casing soil.

High-throughput sequencing revealed the top four fungal phyla detected in the five soil samples, namely *Ascomycota*, *Basidiomycota*, *Mucoromycota*, and *Chytridiomycota* (Figure 8A). *Ascomycota* showed the highest relative abundance, which increased gradually from 41.85% at A1 to 91.31% at A5. *Basidiomycota* showed the highest relative abundance of 36.04% at A3. The relative abundance of *Mucoromycota* was the highest (54.31%) at A1 and subsequently decreased gradually. *Chytridiomycota* exhibited a relative abundance of ≤3.62% during the casing cultivation process, and all other identified fungi had a relative abundance of ≤1.09%.

At the genus level, seven of the top nine genera were unclassified (Figure 8B). The genus *Aspergillus* had the highest relative abundance, ranging from 0.03 to 46.12%. Other genera exhibited the following relative abundances: an unclassified genus of the class *Leotiomycetes*: 0.61–35.28%, an unclassified genus of the order *Agaricales*: 0.13–34.16%, an unclassified genus of the family *Pezizaceae*: 0–15.61%, *Cladosporium*: 0.11–29.43%, an unclassified genus of the order Hypocreales: 2.05–12.48%, an unclassified genus of the order *Pezizales*: 0.11–15.13%, an unclassified genus of the order *Mortierellales*: 2.09–9.26%, an unclassified genus of the order *Onygenales*: 0–19.48%. All other fungi genera had a relative abundance of 16.30–63.26%.

**Figure 9:**
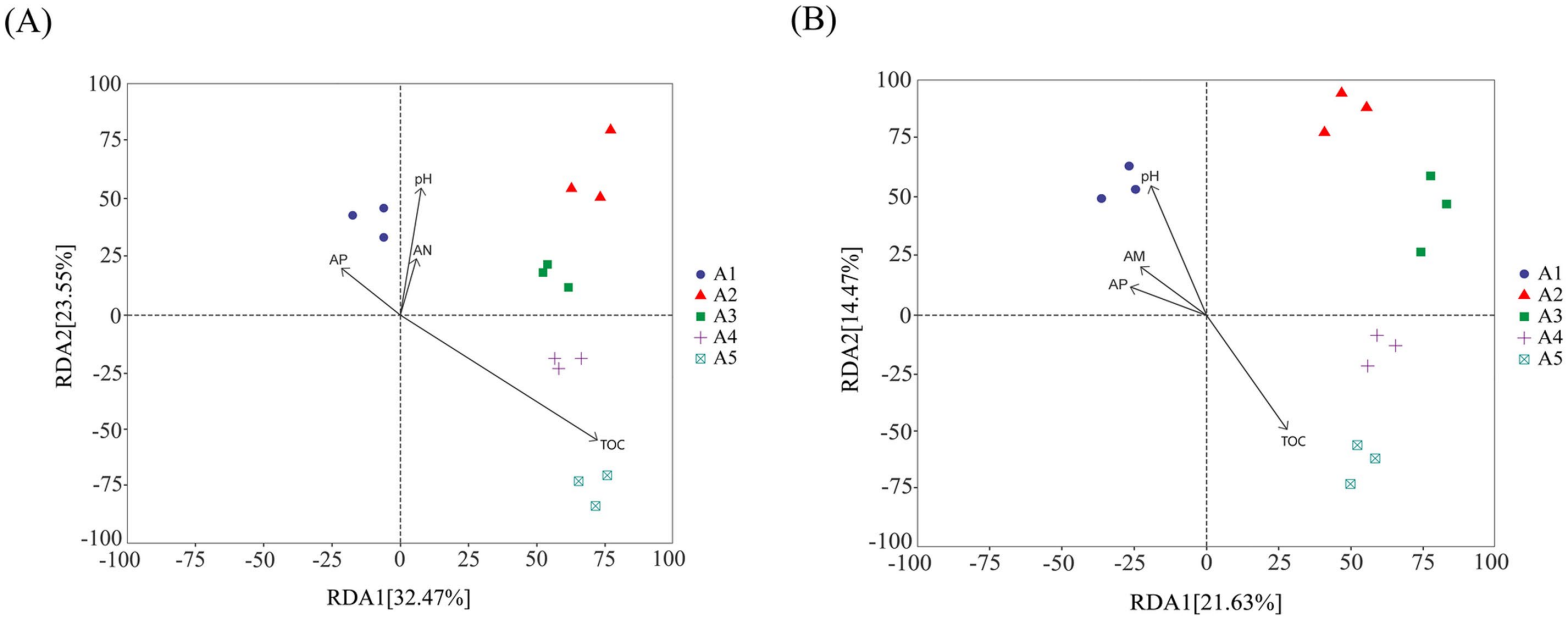
Heatmap of soil microorganisms at the genus level. **(A)** Heatmap of bacteria in soil; **(B)** Heatmap of fungi in soil.

The top 35 bacterial and fungal taxa were selected based on abundance and clustered at the genus level according to their abundance in each sample. A heatmap was generated to visualize species concentration across each sample. For bacterial communities at the genus level, the relative abundance of soil bacteria differed across five stages (Figure 9A). Unclassified *Microscillaceae*, unclassified *Vicinamibacterales*, WD2101 soil group, *Pirellula*, *Gemmata*, unclassified *Pedosphaeraceae*, unclassified *Alphaproteobacteria*, *Steroidobacter*, *Gaiella*, unclassified *Gemmataceae, Dongia, Pseudarthrobacter,* and unclassified *Pirellulaceae* exhibited the highest relative abundance during the casing stage; *Pedosphaeraceae*, unclassified *Gemmatimonadaceae, Sphingomonas*, *Actinobacteria* (IMCC26256), *Haliangium* reached their highest relative abundances in the mycelial stage; *Nocardioides, Aeromicrobium, Lysobacter*, unclassified *Nocardioidaceae, Streptomyces*, lamia, unclassified Acidimicrobiia, and Gitt-GS-136 showed the highest relative abundance during the primordial stage; KD4-96, unclassified *Comamonadaceae* reached peak relative abundance in the fruiting stage; *Lechevalieria*, MB-A2-108 (*Actinobacteria*), *Pseudomonas*, and unclassified *Methylophilaceae* exhibited the highest relative abundance during the harvesting stage.

Similarly, for fungal communities at the genus level, the relative abundance of soil fungi differed across different stages (Figure 9B). Unclassified *Agaricales*, unclassified *Eurotiomycetes*, unclassified *Stereaceae*, *Penicillium, Mucor*, and unclassified *Mortierellales* had the highest relative abundance in the casing stage but decreased in the later stages; *Fusarium*, unclassified *Hypocreales*, unclassified *Ceratobasidiaceae*, and unclassified *Sordariomycetes* showed highest relative abundances in the mycelial stage; unclassified *Ascodesmidaceae*, *Mortierella*, unclassified *Agaricomycetes*, unclassified *Leotiomycetes*, unclassified *Chytridiomycetes*, unclassified *Pezizales*, unclassified *Auriculariales*, unclassified *Pezizaceae*, and *Chaetomium* showed the highest relative abundance during the primordial stage; unclassified *Pleosporales*, Haplotaxida, unclassified *Filobasidiaceae, Cladosporium*, unclassified *Pleosporaceae*, *Tetracladium*, and *Neophaeosphaeria* reached peak relative abundances in the fruiting stage; *Aspergillus*, unclassified *Onygenales*, unclassified *Saccharomycetales*, unclassified *Ascomycota*, and BOLA868 had the highest relative abundance during the harvesting stage.

#### 3.4.4 SparCC-based co-occurrence network analysis of soil microorganisms

**Figure 10:**
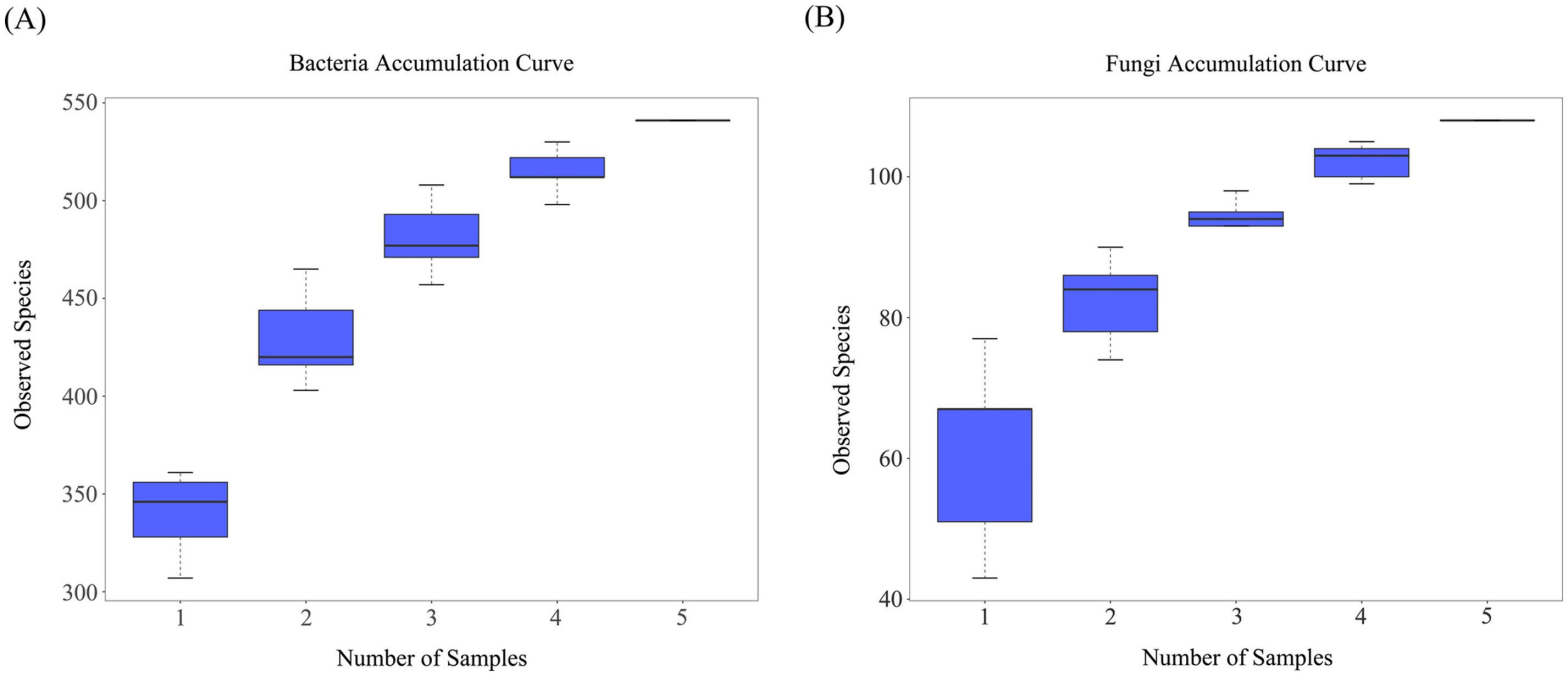
Co-occurrence networks of bacterial **(A)** and fungal **(B)** genera at five cultivation stages of *Oudemansiella raphanipes.*

The SparCC-based co-occurrence network showed that the interactions between some microbial groups (including bacteria and fungi) were altered at different stages of *O. raphanipes* cultivation (Figure 10). The number of nodes between different fungal genera was smaller than that between different bacterial genera, indicating that the relationship between fungal genera was less complex than that between bacterial genera. In the bacterial groups, MND1 and *Vicinamibacteraceae* were positively correlated at the stages A1, A2 and A5. *Gemmatimonadaceae* had complex relationships with other bacteria in different cultivation stages, and the complexity was at the highest level.

At the later stage of cultivation, the number of nodes between fungal genera decreased, indicating that the associated complexity decreased. *Mucor* showed different degree of complexity in different stages of cultivation. The complexity of *Aspergillus* gradually increased, and a new positive and negative correlation was established, which may be caused by the gradual increase in soil pollution.

## 4 Discussion

As a soil-borne wood-decay fungus, *O. raphanipes* strongly depends on soil. The microbial metabolic processes, such as oxidation, nitrification, ammonification, nitrogen fixation, and sulfidation, of the abundant soil microorganisms promote the decomposition and transformation of soil organic matter. Ke et al. (2020) found that using different soil types for casing affects the primordium formation rate, fruiting body traits, mushroom yield, and biological conversion efficiency of *O. raphanipes*. In this study, five growth stages of the soil used for casing *O. raphanipes* were selected as the research project. Soil enzyme activity and physicochemical properties were determined, and high-throughput sequencing combined with various ecological and statistical analyses was used to study the characteristics of microbial communities in the soil at different casing stages and their influencing factors.

The results showed that during *O. raphanipes* cultivation, as the number and activity of microbial communities in the soil changed, sucrase activity gradually increased to maintain soil metabolic balance (Plassart et al., 2019; Zhao et al., 2021). Urease activity decreased with the cultivation process, and catalase activity consistently intensified. This may be influenced by the activity of soil microorganisms and its close connection to the transformation, absorption, and utilization of fundamental nitrogen elements in the soil (Lu and Li, 2020).

Soil plays an essential role in ecosystems and is a sensitive indicator of soil biological traits (Archana et al., 2018; Zhu, 2020). In the present study, the number and species of microbial communities were significantly increased during the cultivation process. This could be because the metabolites produced by the growth of fruiting bodies during the cultivation process changed the composition of the soil nutrients and affected the diversity of microbial communities. Soil pH was significantly decreased during the harvesting stage, whereas the organic matter content was increased, with no significant changes in the AN and AP. This may be owing to some secretions or metabolites in the growth process of dominant microorganisms in the soil, including *O. raphanipes*, which caused corresponding changes in the physicochemical properties of the soil.

Soil TOC, pH, AP, and AN had different effects on bacterial and fungal communities. Bacterial community in soil were most affected by the TOC, while fungal community were most affected by pH. It may be attributed to the fact that these two factors were more specific to one or several types of bacteria and fungi in the soil. There were more *Azotobacter* in soil samples at stage A1, which may be the reason why AN had a significant effect on samples A1. The TOC was positively correlated with the changes of fungal community in many soil samples, indicating that the growth of fungi was highly dependent on organic matter, and abundant organic matter could promote the growth of fungi. However, in addition to the physical and chemical properties of the soil, the factors of *O. raphanipes* at different stages of cultivation should also be included in the redundancy analysis between microbial communities before comprehensive analysis.

In the bacteria found in the soil in this study, the family *Gemmatimonadaceae* belongs to the phylum Firmicutes, which can improve the stress resistance of organisms (Timmusk et al., 2011); the genus MND1 has shown a potential correlation with the resistance against bacterial wilt in mulberry trees (Wu et al., 2020). In practical applications, whether *Gemmatimonadaceae* and MND1 bacteria can be artificially introduced during cultivation to become dominant communities in casing soils to control bacterial contamination and achieve biocontrol effects remains to be studied. The phylum Ascomycota was the dominant fungal community in the casing soil for *O. raphanipes*, with a relative abundance of 0.41–0.91, peaking during the harvesting stage. This group may also contain beneficial microorganisms that positively affect *O. raphanipes* growth. These findings are consistent with those of previous studies on the casing fungal communities of edible mushrooms such as *Agaricus bisporus*, *Agaricus sinodeliciosus*, and *Morchella* (Siyoum et al., 2016; Zhou et al., 2017; Qin et al., 2022).

At specific cultivation stages, different bacteria and fungi reach peak relative abundance, suggesting that these microorganisms may play unique roles in the growth and development of *O. raphanipes* during these periods. An analysis of the microbial community structure in the casing soil showed that the bacterial communities in the soil during the primordial and fruiting stages were similar, and the internal differences in the fungal communities during the mycelial, primordial, and fruiting stages decreased. It is speculated that the soil environment during these stages may be suitable for the growth of certain microorganisms, which could support primordium and fruit body formation.

Casing soil provides an essential environment for transitioning edible mushrooms from the nutritional stage to the reproductive stage. Soil physicochemical properties are crucial factors affecting microbial communities and exhibit dynamic changes, influencing microbial diversity and structure, as well as the yield and quality of fruiting bodies. RDA indicated that soil pH, organic matter, AN, and AP significantly affect the bacterial community structure. Soil pH and organic matter significantly affected fungal communities. Further studies are required to understand how these physicochemical properties affect *O. raphanipes* quality through microorganisms and to identify specific microbial species that may support primordium and fruit body development, which could help unravel the microecological mechanisms in *O. raphanipes* casing soil, providing insights into large-scale production of *O. raphanipes*.

## 5 Conclusion

In this study, soil samples were collected from an artificial *O. raphanipes* greenhouse in Taiyuan City, Shanxi Province, China, and the results had guiding significance for *O. raphanipes* local cultivation. However, considering major regional differences in soil factors, samples should be collected continuously from greenhouses in different regions in the future. In addition, other research methods should be adopted to explore the mechanisms underlying microbial community structure and *O. raphanipes* growth and development further.

## Data Availability

The datasets presented in this study can be found in online repositories. The names of the repository/repositories and accession number(s) can be found in the article/Supplementary material.
