# Supplementary material for: Physicochemical property and microbial community characteristics of the casing soil for cultivating Oudemansiella raphanipes
Source: Front Microbiol. 2024 Dec 5;15:1495168. doi: 10.3389/fmicb.2024.1495168 (PMC11655482; doi:10.3389/fmicb.2024.1495168)
Supplement: Supplementary file 1 [file Data_Sheet_1.pdf]

Supplementary Material

1 Supplementary Figures and Tables

1.1 Supplementary Figures

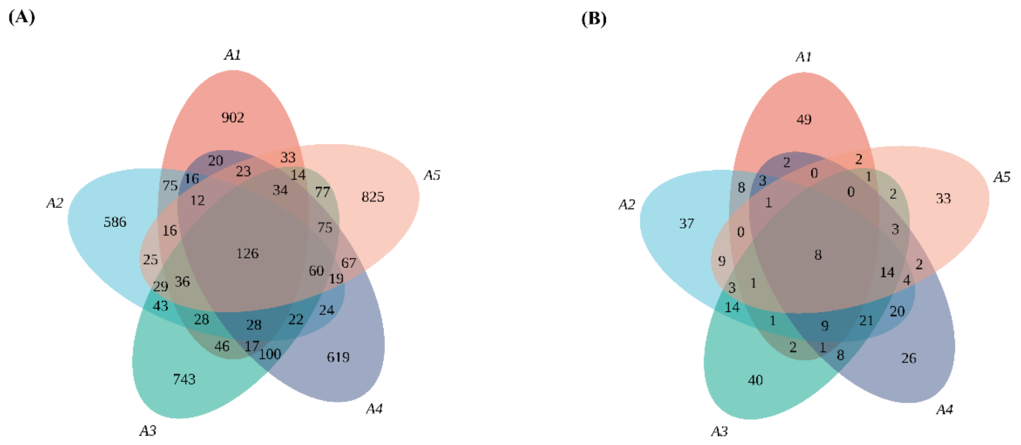

Supplementary Figure 1. Venn plot of OTU differences among cultivation stage samples. (A) Bacterial Venn plot of OTU. (B) Fungal Venn plot of OTU. A1: casing stage; A2: mycelial stage; A3: primordial stage; A4: fruiting stage; A5: harvesting stage.

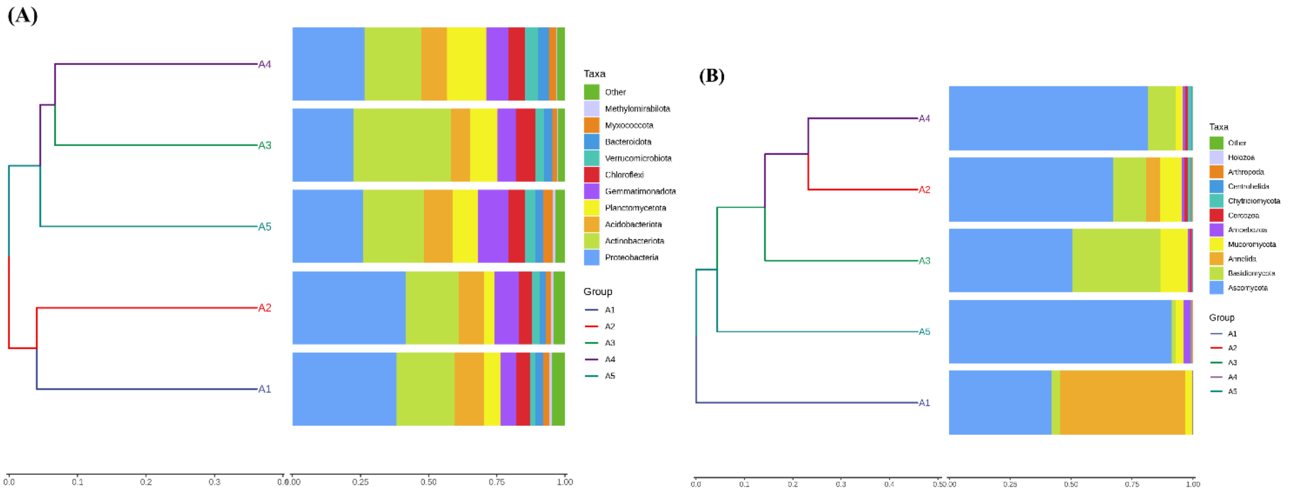

Supplementary Figure 2. Hierarchical clustering of microorganisms in cultivation stage samples at phylum level. (A) Hierarchical clustering of bacteria. (B) Hierarchical clustering of Fungi. A1: casing stage; A2: mycelial stage; A3: primordial stage; A4: fruiting stage; A5: harvesting stage.

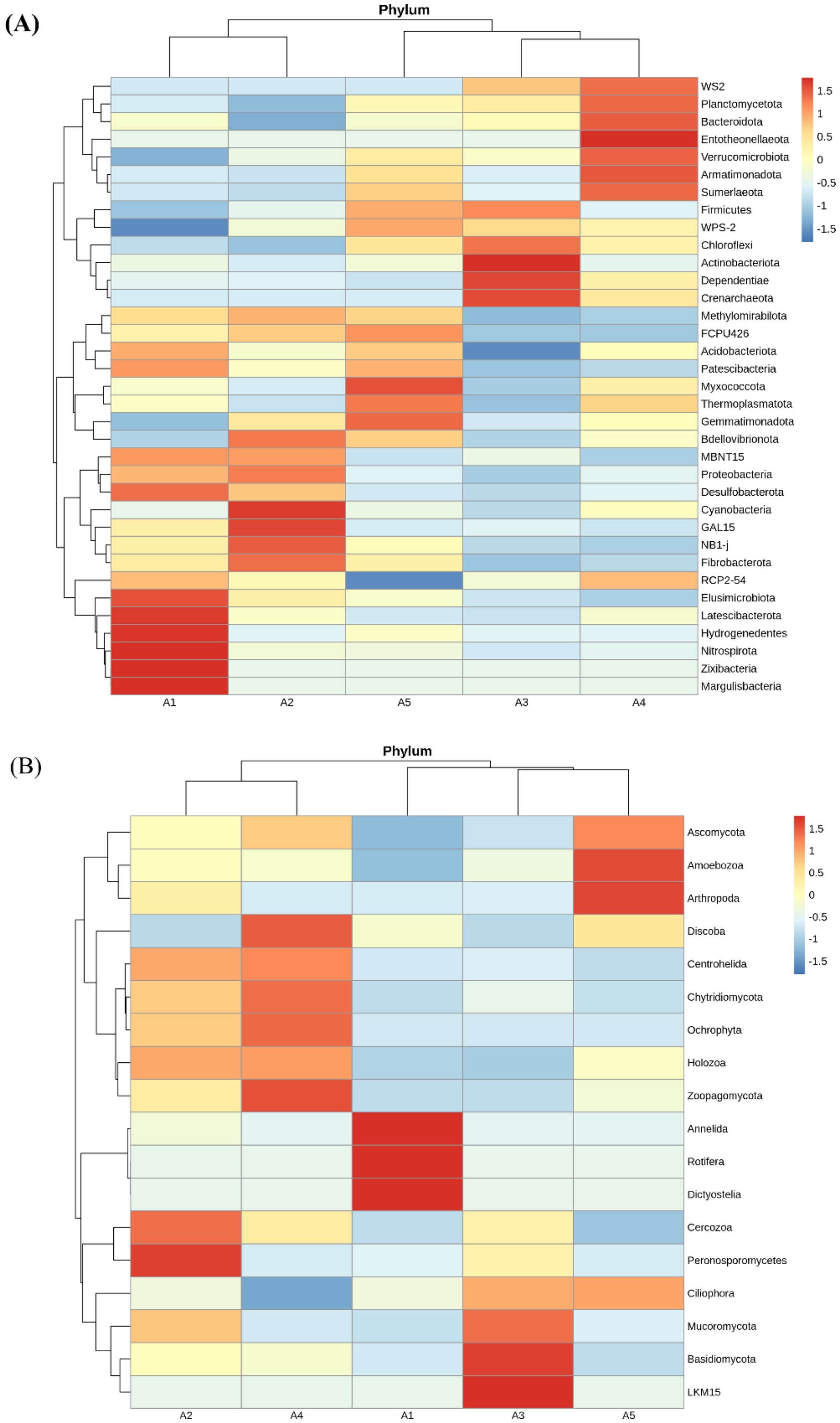

Supplementary Figure 2. (A) Cluster analyses between bacteria communities and cultivation stages at the phylum level. (B) Cluster analyses between fungal communities and cultivation stages at the phylum level. A1: casing stage; A2: mycelial stage; A3: primordial stage; A4: fruiting stage; A5: harvesting stage.

1.2 Supplementary Tables

Supplementary Table 2. Sequencing data

Sample		Raw Reads	Raw Bases	Clean Reads	Clean Bases	Clean Ratio	Q20	Q30	GC Content
Bacteria	A1	145144	32658286	143724	32331075	99.02%	95.96%	89.60%	56.77%
	A2	114452	25753532	113270	25480937	98.97%	95.73%	89.05%	56.82%
	A3	155848	35067827	154430	34741968	99.09%	96.11%	89.80%	57.28%
	A4	115770	26049107	114582	25776032	98.97%	95.70%	89.07%	57.15%
	A5	135032	30383185	133702	30076790	99.02%	95.89%	89.42%	57.32%
Fungi	A1	385006	85670632	379500	84436662	98.57%	94.28%	86.65%	44.70%
	A2	295404	65732428	290748	64625576	98.42%	94.06%	86.25%	40.69%
	A3	394484	87777977	388426	86416847	98.46%	94.01%	86.16%	40.88%
	A4	376326	83737754	370526	82440663	98.46%	94.18%	86.45%	40.34%
	A5	343212	76370269	338268	75259956	98.56%	94.07%	86.30%	40.78%

Supplementary Table 2. α diversity

Sample		Observed species	Shannon	Simpson	Pielou	Chao1	PD_whole_tree
Bacteria	A1	1426	6.623744	0.997546	0.912031	1435.567	50.7453
	A2	1145	6.286529	0.993013	0.892572	1165.663	41.50182
	A3	1478	6.722337	0.997851	0.921064	1492.583	43.95821
	A4	1262	6.652442	0.997656	0.931655	1270.966	44.95935
	A5	1471	6.803506	0.998316	0.932792	1486.68	47.77334
Fungi	A1	88	1.659135	0.654805	0.370563	88	3.014252
	A2	153	3.640754	0.951336	0.723745	153	4.525206
	A3	128	3.111196	0.911171	0.641215	128	3.188399
	A4	122	2.838005	0.884756	0.590756	122	3.049211
	A5	83	2.085233	0.748321	0.471896	83	2.835505
